# The effect of sandblasting and acid etching on survival rate of orthodontic miniscrews: a split-mouth randomized controlled trial

**DOI:** 10.1186/s40510-020-00347-z

**Published:** 2021-01-07

**Authors:** Saeid Foroughi Moghaddam, Amir Mohammadi, Ahmad Behroozian

**Affiliations:** Department of Orthodontics, Faculty of Dentistry, Tabriz University of Medical Sciences, Golgasht Avenue, Tabriz, Iran

**Keywords:** Orthodontic mini screws, Sandblasting, Survival rate, Surface roughening

## Abstract

**Background:**

The aim of this study was to investigate the effect of surface roughening and acid etching on clinical success rate and removal and insertion torque of orthodontic miniscrews.

**Materials and methods:**

Sixty-two orthodontic miniscrews (Jail Medical Corporation, Seoul, Korea) with the same design and dimensions (10-mm length, 2-mm diameter) are divided into two (sandblasted and acid-etched versus control) groups. The sample of the study was 31 patients whose miniscrews were needed for en masse retraction of the upper six anterior teeth. In this split-mouth study, the miniscrews were placed in the attached gingiva between the second premolar and the first molar. The side (left or right) was selected randomly. The miniscrews were loaded 6 weeks after insertion, and the patients were followed up after 3, 6, 10, 14, and 18 weeks and then for 4 weeks interval. Chi-square, correlation, and independent *t* tests were done using SPSS ver24 to interpret the data.

**Results:**

The survival rate was 90.3% and 83.9% for the sandblasted and acid-etched versus the control group, respectively. The difference in survival rate was not statistically significant (*p* > 0.05). Removal torque was higher for the sandblasted group (*p* < 0.05). Younger patients showed less survival rate (*p* < 0.05) in both groups. Insertion side, namely, left or right, was not statistically significant.

**Conclusions:**

Although sandblasting increased removal torque, it did not influence the survival rate of orthodontic miniscrews significantly.

**Supplementary Information:**

The online version contains supplementary material available at 10.1186/s40510-020-00347-z.

## Background

Providing the adequate anchorage is necessary in many orthodontic treatments to control the reciprocal force of tooth movement [1]. Many techniques and appliances have been designed for preserving anchorage, such as transpalatal arch, Nance appliance, extra-oral anchorage, engagement of multiple teeth in the anchorage unit, and applying differential moments. Miniscrews have been introduced in orthodontics as a skeletal anchorage that can tolerate reaction forces applied to the teeth [2] and as a temporary anchorage device which does not require invasive surgery and has overcome many of the issues associated with the larger devices. In addition, they do not need laboratory process or large armamentarium to use. Although preliminary data was promising, there are some questions about optimal surface characteristics than remain subjects for further investigation [3].

The success rate of orthodontic miniscrews varies from 60 to 91%. The failure of micro-implants due to loss of stability is a multifactorial problem [4, 5]. Although partial osseointegration of mini-implants may improve the stability [4, 6], the stability of orthodontic miniscrews depends on mechanical locking of the threads rather than osseointegration [7].

Since surface topography could affect cell growth and orientation [3] and surface treatment can increase the interdigitation between miniscrew and bone, working on this phenomenon can be a possible solution for increasing the success rate of the miniscrews [8]. In addition, removal torque can reflect the quality of the interface between miniscrew and bone, so it can be considered as a parameter in the evaluation of the temporary anchorage devices [9]. Park et al. investigated the effect of acid etching on a success rate. Although it was a split-mouth study, the site of insertion and the force application method were not identical for all of the patients [10].

The purpose of the present study was to investigate the effect of surface roughening by sandblasting and acid etching on survival rate and removal and insertion torque of orthodontic miniscrews in a split-mouth study. To our knowledge, this is the first split-mouth controlled trial with identical sites and force application that has addressed this issue on human subjects.

## Materials and methods

### Study population

In this single-blinded, split-mouth randomized clinical trial, 31 orthodontic patients (8 male, 23 female; mean age of 18.5 years) whose treatment plan included the use of orthodontic miniscrews bilaterally between the upper first permanent molar and the second premolar were selected from patients referring to the author’s private office Tabriz, Iran. The treatment plan of all the patients included en masse retraction of the upper six anterior teeth. The patients suffering from bone diseases like osteopetrosis, osteoporosis, or other systemic conditions that affects bone metabolism were excluded. All persons gave their informed consent prior to their inclusion in the study.

### Samples

**Fig. 1:**
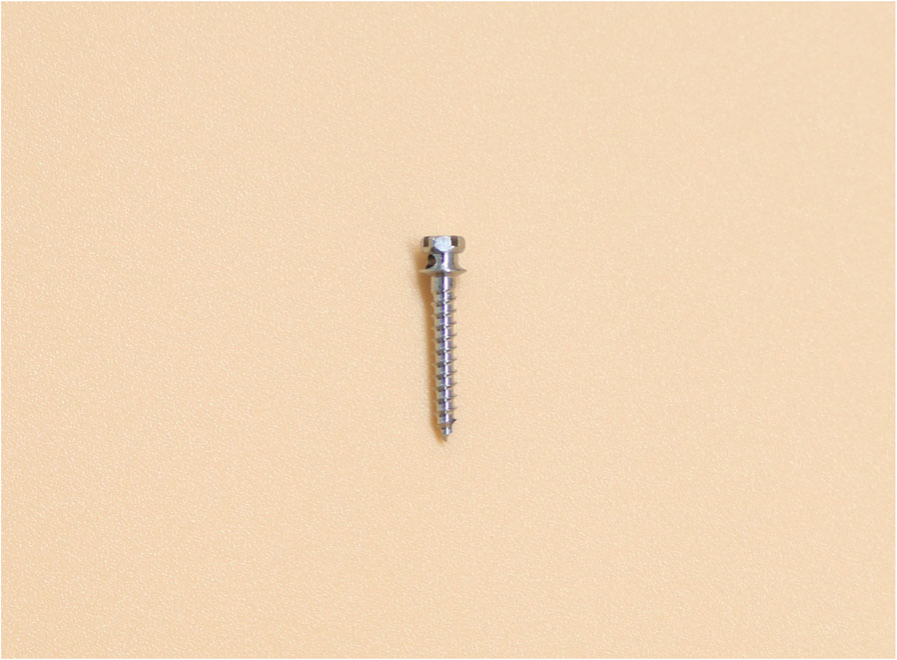
Orthodontic miniscrews

The sample included 62 miniscrews with the same shape and dimension (Dual-Top Anchor system, 1.6-mm diameter, 10-mm length, self-drilling style, Jeil Medical Co, Seoul, Korea) (Fig. 1) which was divided into two groups: the sandblasted acid-etched (SAE) and the control group.

### Sandblasting and acid-etching process

**Fig. 2:**
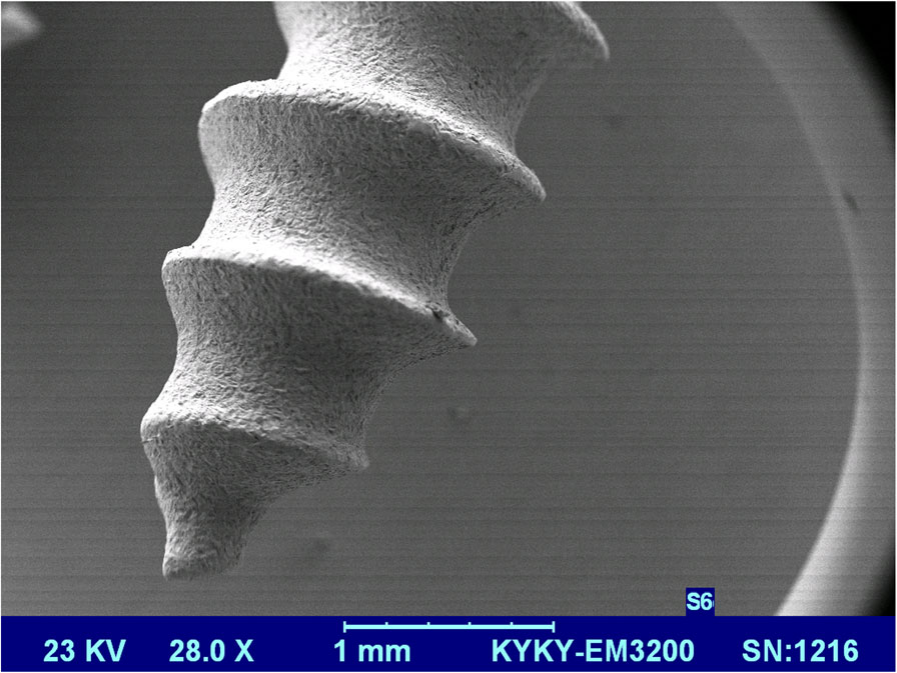
Scanning electron microscope images of miniscrews in the sandblasted and acid-etched group

**Fig. 3:**
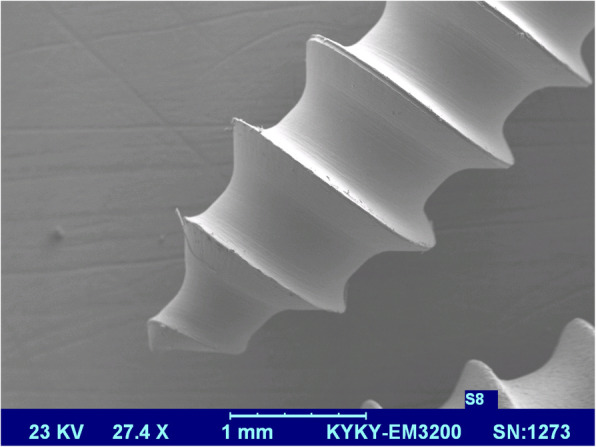
Scanning electron microscope images of miniscrews in the control group

Miniscrews were blasted first with alumina particles of grain size 250 μm in the pressure of 4 MPa and then rinsed with acetone, 75% ethanol, and distilled water for 15 min in an ultrasonic cleanser, then were placed in 0.11 HF mol/l and 0.09 mol/l HNO_3_ solution in 25 °C temperature for 10 min. After etching, miniscrews were dried in an oven with 50 °C for 24 h; one of the SAE and control miniscrews were checked by a scanning electron microscope (SEM, Philips 515, Philips, Eindhoven, Netherlands) to confirm the sandblasting process (Figs. 2 and 3).

### Clinical process

**Fig. 4:**
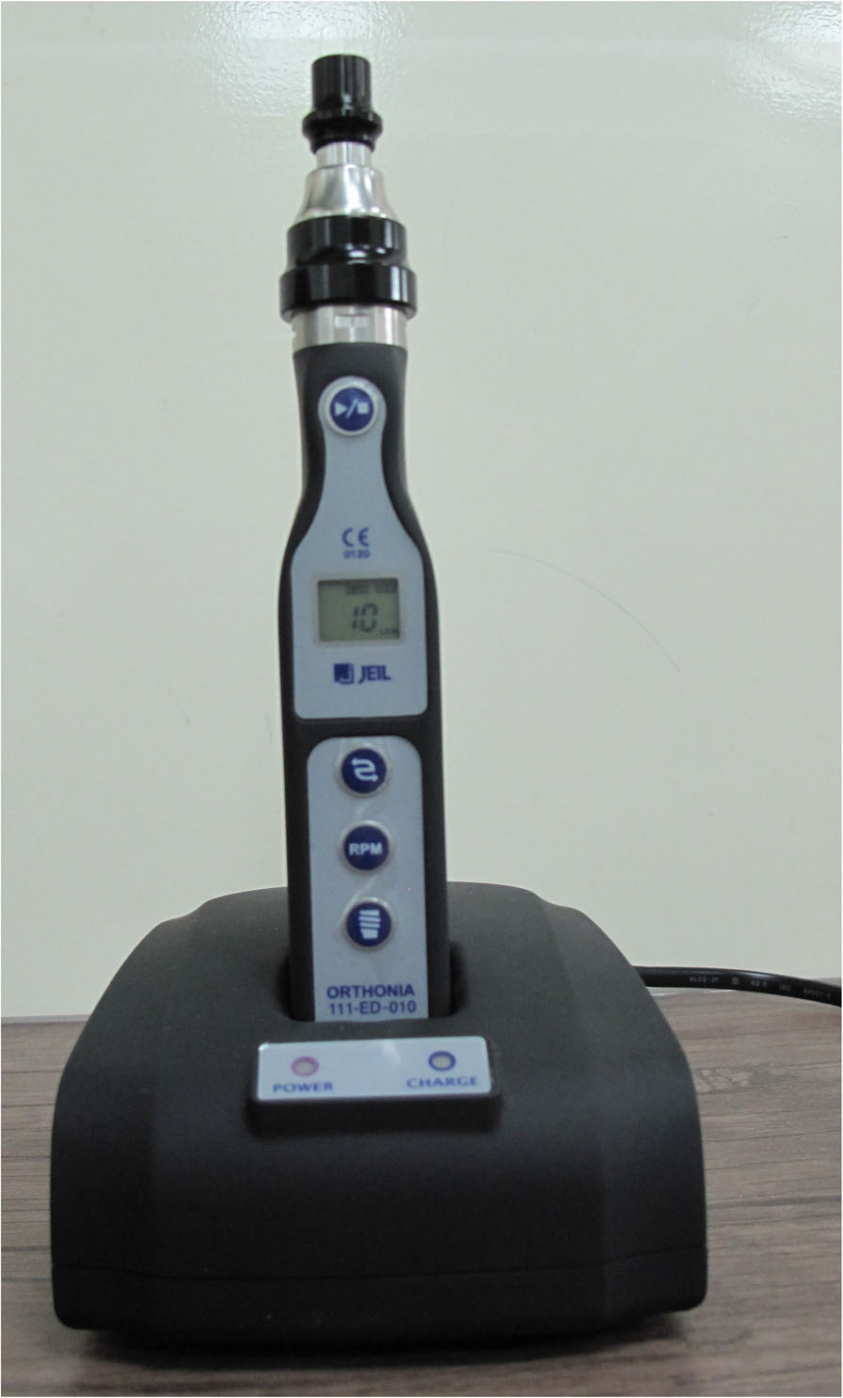
Orthodontic appliance was used to measure insertion torque

**Fig. 5:**
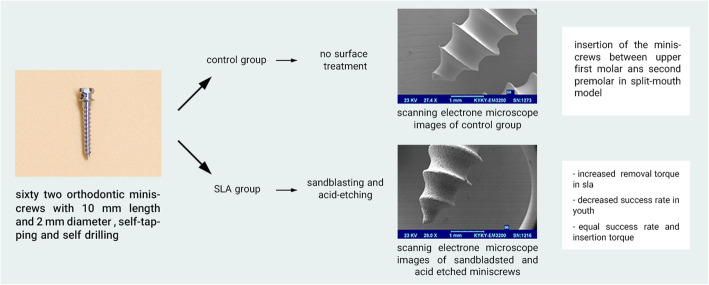
Visual abstract

Informed consent was obtained from patients before surgery. All surgical process were performed by the senior clinician. After local anesthesia, the miniscrews were inserted into the buccal attached gingiva between the first permanent molar and the second premolar, without predrilling or incision using an Orthonica (straight type, Jeil Medical Co, Seoul, Korea) to measure insertion torque (Fig. 4). A pair of miniscrews (SAE and control) were allocated for each patient (Fig. 5). The side in which SAE or control miniscrew were placed, namely, left or right, was determined by flipping a coin. The patients with the odd code in the registration list received SAE miniscrew in the left and even codes received it in the right. The senior orthodontist generated the random allocation sequence, enrolled the participants, and assigned the participants to the interventions. The clinician was aware of the type of the miniscrew because of the dull appearance of the SAE miniscrews, but the patient was not aware of that. The upper six anterior teeth were ligated on 19 × 25 stainless steel wire, and 6 weeks after insertion, a traction force of about 250 g was applied with the use of a nickel-titanium (Ni-Ti) coil spring (Ormco, Orange, Calif) from the miniscrew to the canine. The patients were followed up in 3, 6, 10, and 14 weeks and then in 4 weeks interval, and the mobility of the miniscrews was checked. The failure was defined as the mobility of the miniscrews that precludes its clinical performance or more than 1 mm mobility. The percentages of the miniscrews of each group that were useful up to the end of the treatment were considered as success rate, and the time between insertion and failure point was considered as survival time. At the end of the space closure, the miniscrew was removed and the removal torque measurement was done (NSK Ltd. Surgic XT Implant micromotors, Tokyo, Japan).

### Statistical methods

Statistical analysis was performed with SPSS software, version 24.0 (SPSS, Chicago, IL). *p* < 0.05 was considered significant. The influence of the clinical parameters on the survival rate and removal and insertion torque of the mini-implants was also evaluated using chi-square exact test and independent *t* test, respectively. Fisher’s exact test was performed so that differences in the success rate between groups could be examined. The correlation tests also were done to find the relationship between the criteria.

## Results

### Success rate

For each group, all participants (*n* = 31) were randomly assigned, received intended treatment, and were analyzed for the outcome. The overall success rate was 87.09% (54 of 62 miniscrews) and 87.50% of the failures happened in the first 4 months. There was no statistically significant difference in success rate according to sex and side (Table 1). The young patients showed significantly lower success rate compared to the adults. Although the success rate was higher for the SAE compared to the control group, the difference was not significant. No significant difference was noted in success rate according to the amount of insertion torque (*p* < 0.05).

**Table 1:** The success rate (%) of orthodontic miniscrews

	Orthodontic miniscrew success rate (%) (success number/total number)			
Criteria	Patients	Success rate	*p* value	Sig.
Age	Below 15 years	66.66% (12/18)	0.025	*
	Above 15 years	95.45% (42/44)		
Sex	Male	93.75% (15/16)	0.35	NS
	Female	84.78% (39/46)		
Type	SAE	90.32% (28/31)	0.44	NS
	Control	83.87% (26/31)		
Side	Left	12.90% (27/31)	1.00	NS
	Right	12.90% (27/31)		

SAE indicates sandblasted and acid-etched miniscrews

*Sig.* significance, *NS* not significant

*p < .05

### Insertion torque

No significant difference was reported between the insertion torque of SAE and control (Table 2). Independent *t* test showed no difference in insertion torque according to sex, gender, and side (*p* < 0.05). Correlation tests found no relationship between amount of insertion and removal torque of the miniscrews (*p* < 0.05). There was no significant difference between the insertion torque of failed or of successful miniscrews (*p* < 0.05).

**Table 2:** Insertion and removal torque of orthodontic miniscrews in the study groups

	Insertion torque (Ncm) Range (mean ± SD)	*p* value	Sig.	Removal torque (Ncm) Range (mean ± SD)	*p* value	Sig.
SAE (*n* = 31)	5–30 (12.10 ± 6.295)	0.83	NS	10–30 (15.71 ± 5.563)	0.001	*
Control (*n* = 31)	5–25 (12.42 ± 5.755)			5–10 (8.08 ± 2.481)		

SAE indicates sandblasted and acid-etched miniscrews

*SD* standard deviation, *Sig.* significance, *NS* not significant

*p < .05

### Removal torque

SAE reported significantly higher removal torque compared with control (Table 2). Since the failed miniscrews loose themselves, the removal torque was not measured for failed miniscrew and the comparison of the removal torque of failed and successful miniscrews was not done. Independent *t* test showed no difference in removal torque according to sex, gender, and side (*p* < 0.05).

## Discussion

Many researchers deal with the retention and survival rate of the orthodontic miniscrews. This human split-mouth clinical controlled study aimed to investigate the effect of the surface treatment with sandblasting and acid etching on clinical performance of the orthodontic miniscrews.

The overall success rate was 87.09% (54 of 62 miniscrews) that was similar to the rates previously reported [11]. Also, a meta-analysis by Beltrami et al. reported that the mean weighted overall success rate was 86.75 ± 8.48% [12].

This was the first split-mouth human study with exactly matched cases which evaluated the effect of sandblasting and acid etching on the stability of orthodontic miniscrews. The advantage of the split-mouth study is that the case and control intervention is done in the same patient. Therefore, it removes a lot of inter-subject variability from the estimated treatment effect. This comes more important in our study where the properties of soft and hard tissues of the different patients may influence the success rate of the screws. Park et al. investigated the effect of acid etching on the success rate of miniscrews [10]. They did not use sandblasting, and despite the split-mouth design, the site of miniscrew insertion was different in the patients: some of them in the maxilla and the others in the mandible. Also, force vector and application were not identical for all of the patients, some for en masse retraction, and others for intrusion or even distalization. Several studies has addressed this issue on the month or even long bones of different animals like dogs [13], rabbits [9], and minipigs [14] and even artificial bone [15]. But individual host-related factors of specimens which include bone quantity and quality, inflammation of the peri-implant tissue, proximity of the miniscrew to the adjacent teeth and cortical to trabecular bone ratio as well as the overall morphology of the patient can play a role in the survival of the orthodontic miniscrews [6, 12, 16]. Since soft tissue characteristics and bone quality can influence the results of the study, the outcomes of the animal studies cannot be simply generalized to the human. The same is true about some human studies in which the site of miniscrew insertion or the type of miniscrew were not matched between the case and the control groups [10, 11, 17, 18].

### Sandblasting

Surface roughening can ensue more retention of orthodontic miniscrews via two major mechanisms: first, mechanical interlocking, and second, biological integration. Implant surface properties is one of the main factors influencing integration with bone [19]. Surface roughness can play a role in bone integration and fibroblast adhesion and differentiation [20]. Partial osseointegration of orthodontic miniscrews has been suggested by some researchers as a major retention mechanism, but the amount of this osseointegration and its role in success rate of the mini-implants remain controversial [14].

Several methods have been tried by the researchers to treat the surfaces of orthodontic miniscrews like different methods of acid etching [9], anodization, microgroove preparation [13], and different techniques of sandblasting [9]. According to Yadav et al., alumina blast plus acid etching yielded in the highest bone-to-implant contact and torque compared with rival techniques [9], so we used this to create surface roughness. Furthermore, they reported that grit blasted with acid etching is the most hydrophilic surface. Hydrophilicity of the surface is mandatory for protein absorption and cell adhesion [21].

### Force application

According to Liou et al., application of force and the pattern of its application can influence the osseointegration and tissue adaptation pattern around the miniscrew [22, 23]. Kim et al. reported that the properties of surrounding tissue of the miniscrew differs in tension and compression side [13]. So, the investigation of the miniscrews in real clinical situation in terms of force application is of importance and assessing the success rate without applying orthodontic force cannot reflect real clinical situation. Most implant losses is attributed to the strains at the bone-implant interface [24]. On the other hand, even in the usual orthodontic force (i.e., 100–300 gr), the stress concentrates in a small area and increases up to 33 MPa [25] because the dimension of the orthodontic miniscrew is small. Therefore, we applied 250 gr continuous force via NiTi open coil which is clinically useful and in the tolerance range of the orthodontic miniscrews.

### Removal torque

In agreement with Motoyoshi et al., we found that there was not any correlation between insertion and removal torque and higher insertion torque did not yield higher success rate [26], But we found that sandblasting and acid etching increased removal torque. This finding is in agreement with previous studies who reported increased removal torque with surface treatment [9, 19]. They attributed increased removal torque to increased bone-to-implant contact which was shown in the histologic view. Kim et al. proved removal torque as a reliable measure of stability [27]. Microscopic examination showed increased bone-to-implant contact in roughened miniscrew. Others assumed bone-to-implant contact as a marker of osseointegration and concluded that partial osseointegration that takes place in sandblasted miniscrews results in increased removal torque [17, 28]. Although these theories are generally accepted, there are some unanswered questions in this issue: does close contact of bone and implant which is seen in the microscopic view always mean osseointegration? If so, why did in our study removal torque—and probably osseointegration—increased but the success rate did not? Is there any factor more important than osseointegration in success rate of miniscrews in split-mouth matched cases? More studies are needed to answer these questions.

It could be registered as a general trend of direct proportionality between success rate and age [12]. Motoyoshi et al. reported that immediate loading of mini-implants had significantly higher success rate in adults than adolescents [29]. Our study showed lower success rate of orthodontic miniscrews in young patients. This can be attributed to the lower bone density in adolescence, so in young patients, orthodontic miniscrews should be used with caution and some additional anchorage preparation may be considered [30].

### Suggestion and limitation

Although increase of removal torque in sandblasted and acid-etched miniscrews is encouraging, future split-mouth studies with larger sample sizes are warranted to determine if surface treatment would have an effect on the success rate and the clinical performance of the miniscrews in different situations. We suggest other insertion sites of the maxilla and the mandible to try. The inherent limitation of this study includes a lack of histologic evaluation because it was a human study. Lower sample size in some age groups was also a limitation.

## Conclusions

Surface roughness of orthodontic miniscrews by sandblasting and acid etching had no influence on success rate but increased removal torque significantly.


**Additional file 1**

## Data Availability

Not applicable
